# Vitamin C Intake and Pancreatic Cancer Risk: A Meta-Analysis of Published Case-Control and Cohort Studies

**DOI:** 10.1371/journal.pone.0148816

**Published:** 2016-02-09

**Authors:** Yong-Fei Hua, Gao-Qing Wang, Wei Jiang, Jing Huang, Guo-Chong Chen, Cai-De Lu

**Affiliations:** 1 Department of Hepatobiliary and Pancreatic Surgery, Ningbo Medical Treatment Center Lihuili Hospital, Ningbo, 315000, China; 2 Department of Nutrition and Food Hygiene, School of Public Health, Soochow University, Suzhou, 215123, China; Columbia University, UNITED STATES

## Abstract

**Background:**

Observational studies inconsistently reported the relationship between vitamin C intake and risk of pancreatic cancer. We conducted a meta-analysis of published case-control and cohort studies to quantify the association.

**Methods:**

Potentially eligible studies were found on PubMed and EMBASE databases through May 31, 2015. A random-effects model was assigned to compute summary point estimates with corresponding 95% confidence intervals (CIs). Subgroup and meta-regression analyses were also performed to explore sources of heterogeneity.

**Results:**

Our final analyses included 20 observational studies comprising nearly 5 thousand cases of pancreatic cancer. When comparing the highest with the lowest categories of vitamin C intake, the summary odds ratio/relative risk for case-control studies (14 studies), cohort studies (6 studies) and all studies combined was 0.58 (95% CI: 0.52–0.66), 0.93 (95% CI: 0.78–1.11) and 0.66 (95% CI: 0.58–0.75), respectively. The difference in the findings between case-control and cohort studies was statistically significant (*P* < .001). Possible publication bias was shown in the meta-analysis of case-control studies.

**Conclusion:**

There is insufficient evidence to conclude any relationship between vitamin C intake and risk of pancreatic cancer. The strong inverse association observed in case-control studies may be affected by biases (eg, recall and selection biases) that particularly affect case-control studies and/or potential publication bias. Future prospective studies of vitamin C intake and pancreatic cancer are needed.

## Introduction

Globally, pancreatic cancer represents the 4th leading cause of cancer death. Pancreatic cancer has a poor prognosis and high fatality rate, with a 5-year survival rate of less than 5%[1]. Potential risk factors include chronic pancreatitis, cigarette smoking, family history, and diabetes[1, 2]. Dietary factors may also pay a role in the development of pancreatic cancer, but few certainties have been achieved[3].

Oxidative stress is implicated in the pathogenesis of acute and chronic pancreatitis[4]. Both *in vitro* and *in vivo* researches demonstrate an important role of inflammation in the initiation and progression of pancreatic cancer[5]. Vitamin C is a strong antioxidant, and is shown to inhibit preneoplastic lesions in animals’ pancreas[6]. In experimental studies, vitamin C has been found to induce apoptosis, enhance immune function, and protect free radicals-induced DNA damage[3, 7]. Thus, vitamin C intake is biologically plausible to protect against pancreatic cancer.

Recently, an expert panel convened by the Word Cancer Research Fund and the American Institute for Cancer Research concluded that the evidence for a beneficial effect of fruit against pancreatic cancer was limited and inconsistent, and the evidence for vegetable and vitamin C was judged as ‘limited-no conclusion’[8].On the contrary, a more recent meta-analysis of 17 observational studies (13 case-control studies and 4 cohort studies) suggested a significant 29.5% reduction in the risk of pancreatic cancer for high-versus-low vitamin C intake[9]. However, results from this meta-analysis could be limited because of including duplicate publications from the same population[10, 11], analyzing data of dietary vitamin C together with those of circulating vitamin C[12], pooling risk estimates expressed as continuous vitamin C intake in the high-versus-low analysis[13], and missing a number of eligible studies[14–19]. Given the inconsistent evidence, we performed a meta-analysis of published observational studies to better quantify the association of vitamin C intake with risk of pancreatic cancer. Case-control studies and prospective cohort studies were separately analyzed throughout the meta-analysis because they are considerably different in many aspects.

## Methods

### Literature search and selection

A literature search was performed on PubMed and EMBASE databases through May 31, 2015 using the search strategy as follows: (vitamin C OR ascorbate) and (pancreatic cancer OR pancreatic carcinoma OR cancer of pancreas). No language restrictions were imposed. The reference lists of retrieved full-text publications were also carefully screened to identify any further studies. Studies that fulfilled the following criteria were considered eligible: (i) case-control or cohort study design; (ii) vitamin C intake as the exposure of interest; (iii) pancreatic cancer as the outcome of interest; and (iv) odds ratio (OR) or relative risk (RR) or hazard ratio (HR) estimates with corresponding 95% confidence interval (CI) were available.

### Data extraction

The following data were extracted from each included study by use of a standardized data-collection form: the first author’s last name, publication year, country, source of controls (for case-control studies), length of follow-up (for cohort studies), sex of subjects, sample size, source of vitamin C, categorized vitamin C intake and corresponding risk estimates that reflected the greatest degree of adjustment, and variables accounted for in the statistical model. Literature search, study selection and data extraction were carried out independently by two authors (YFH and WJ), with any disagreement resolved by consensus.

### Statistical analysis

In this meta-analysis, the common measure of association was OR in case-control studies, and RR in cohort studies. Results from case-control and cohort studies were separately presented. A random-effects model[20] which considers both within-and between-study variation was assigned to combine study-specific risk estimates. Several studies presented results for different sources of vitamin C intake (total, diet and supplement), and the results for total intake were included in the main analysis. Subgroup analysis were performed according to geographic area, source of control (for case-control studies), sex of subjects, years of follow-up (for cohort studies), number of events, and source of vitamin C.

Considering the distinct cutoffs across studies, we conducted a dose-response analysis with the method of Greenland and Longnecker[21] and Orsini*et al*.[22]. The method requires that the number of cases and controls (or person-years) and the risk estimates with their variance for at least 3 quantitative exposure categories are known. For every study, the median/mean level of the intake for each category was assigned to each corresponding risk estimate. When the median/mean intake per category was not provided, the midpoint of the upper and lower boundaries in each category was assigned as the average intake. If the highest/lowest category was open-ended, we assumed the width of the interval to be the same as in the closest category. Because of limited number of eligible studies in case-control studies[16, 18, 23]., we only conducted the dose-response analysis for prospective cohort studies.

Heterogeneity test was performed using Q and *I*^2^ statistics [24]. For the Q statistic, level of significance was set at *P<*0.1; and for the *I*^2^, a value of <25%, 25–75% and >75% represents little/no, moderate, and considerable heterogeneity. Potential publication bias was investigated with both Begg correlation test and Egger regression test[25, 26],. All statistical analyses were performed using STATA software, version 12.0 (STATA Corp., College Station, TX, USA).

## Results

### Study characteristics

**Fig 1:**
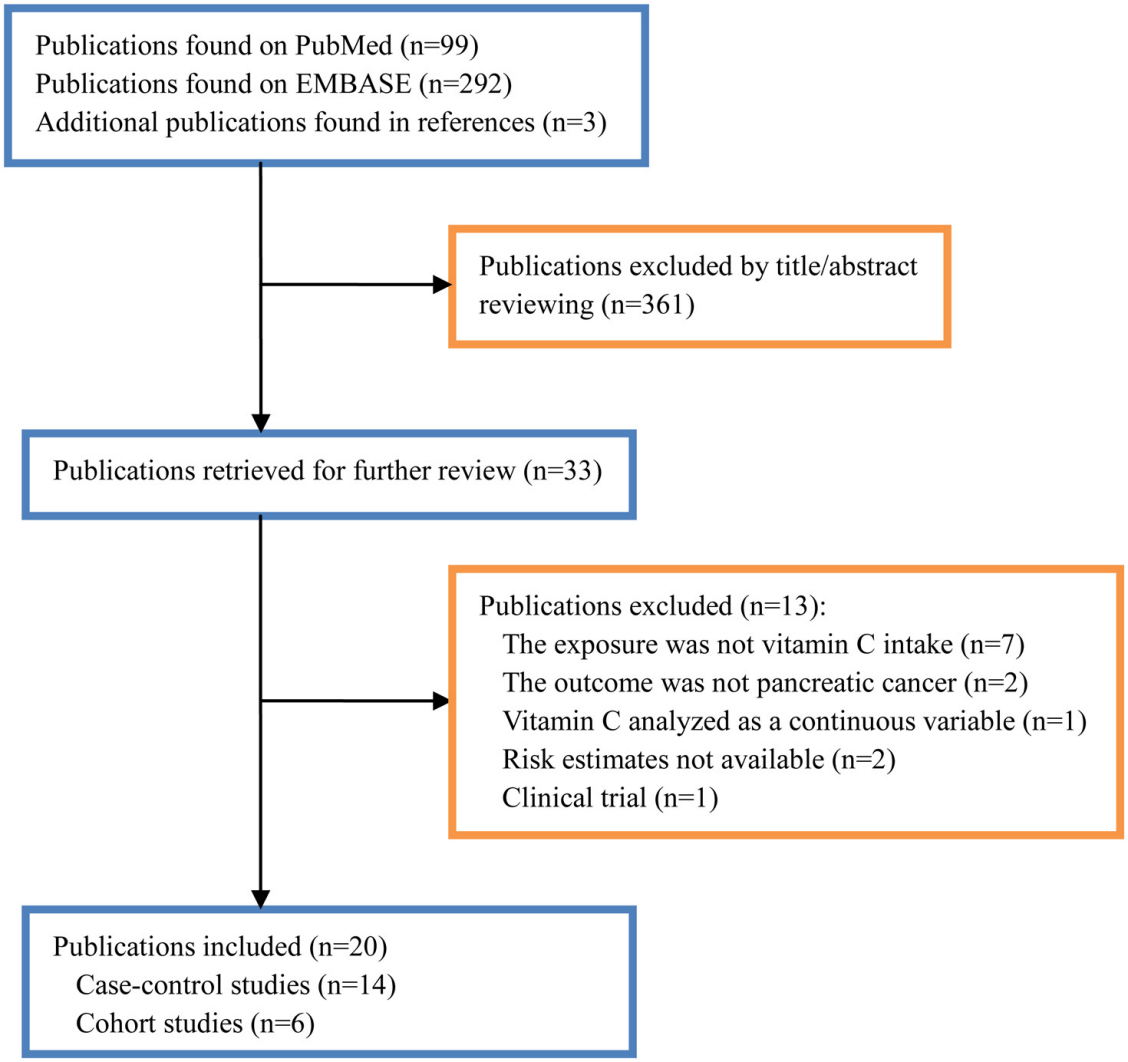
Flow chart of literature search and selection.

A flow chart of study selection is reported in Fig 1. Our final analysis included 14 case-control studies[10, 16–19, 23, 27–34] and 6 cohort studies[14, 15, 35–38]. The 14 case-control studies were published between 1988 and 2013, covering 3,818 pancreatic cancer cases and 10,115 controls. Geographic regions of these studies ranged from North America (*N* = 8), Europe (*N* = 3) to Asia (*N* = 3). Most of the case-control studies selected control subjects from general populations (11/14), and recruited both men and women (12/14). Ten of the 14 studies only reported results for dietary vitamin C, 2 studies merely investigated supplementary vitamin C, and 2 studies involved vitamin C of different sources. The 6 prospective cohort studies were published between 2002 and 2013, with a total of 1,140 cancer events and 278,000 participants. The length of follow-up ranged from 7.1 to 21 years. Four studies only reported results for dietary vitamin C, and 2 studies contained vitamin C of different sources. Four of the 6 studies included both sexes, 1 study included men only, and the remaining 1 consisted entirely of women. Half of the 6 cohorts were from the US, and the others were conducted in Europe. The characteristics of the included case-control studies and cohort studies are, respectively, summarized in S1 and S2 Tables.

### Meta-analysis of case-control studies

**Fig 2:**
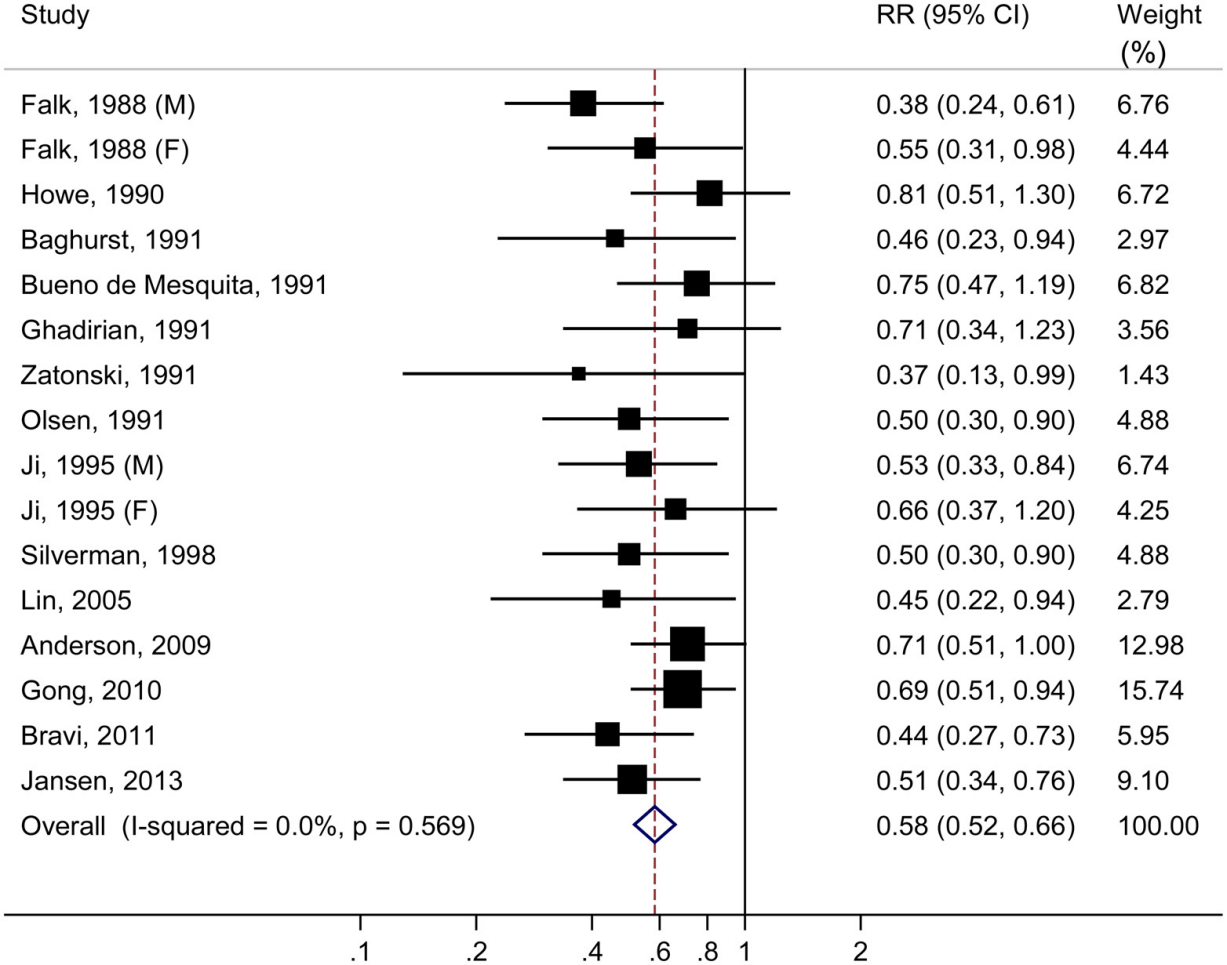
Odds ratios of pancreatic cancer for the highest compared with the lowest categories of vitamin C intake in case-control studies.

The multivariable-adjusted OR of pancreatic cancer from individual case-control studies and all studies combined are reported in Fig 2. All studies showed an inverse association between vitamin C intake and risk of pancreatic cancer, among which 11 reported significant results. Results of the meta-analysis conferred a summary OR of 0.58 (95% CI: 0.52–0.66) for the highest compared with the lowest intake of vitamin C, with no evidence of heterogeneity (*P*_heterogeneity_ = 0.57, *I*^2^ = 0%). Subgroup analysis stratified by predefined study and population characteristics showed consistent results (Table 1). Source of control was found to be an effect modifier (*P*_difference_ = 0.04), with a more pronounced effect among hospital-based case-control studies (OR = 0.46) than population-based ones (OR = 0.63). Vitamin C from diet (OR = 0.58) also showed a stronger protection than supplement (OR = 0.79), but the between-group difference was statistically nonsignificant (*P*_difference_ = 0.29). Dose-response analysis were not performed due to limited number of eligible studies[16, 18,23].

**Table 1:** Results of stratified analysis for case-control studies.

	*N*	OR (95% CI)	*P* for heterogeneity	*I*^2^ (%)	*P* for differences
Area					
North America	8	0.60 (0.51–0.70)	0.36	9.6	0.46
Europe	3	0.54 (0.36–0.83)	0.22	34.7	
Asia-pacific	3	0.53 (0.40–0.72)	0.83	0.0	
Source of control					
Population	11	0.63 (0.55–0.73)	0.79	0.0	0.04
Hospital	3	0.46 (0.37–0.59)	0.73	0.0	
Sex					
Men	5	0.61 (0.44–0.86)	0.02	66.7	0.70
Women	5	0.59 (0.47–0.73)	0.90	0.0	
No. of cases					
≥200	9	0.58 (0.51–0.66)	0.42	2.3	0.90
<200	5	0.59 (0.45–0.79)	0.53	0.0	
Source of vitamin C					
Diet	12	0.58 (0.49–0.69)	0.11	33.4	0.29
Supplement	4	0.70 (0.60–0.82)	0.57	0.0	

### Meta-analysis of cohort studies

**Fig 3:**
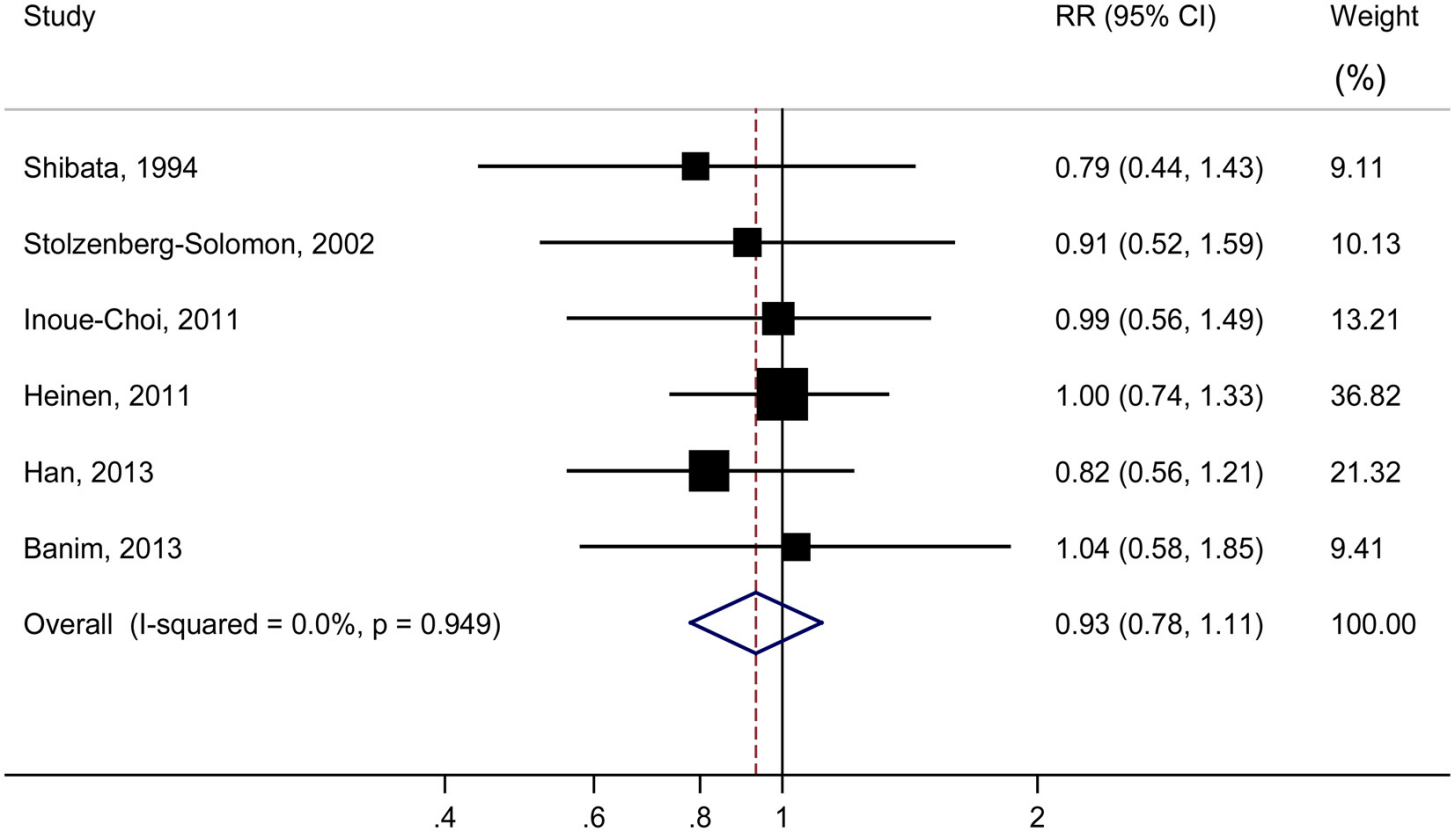
Relative risks of pancreatic cancer for the highest compared with the lowest categories of vitamin C intake in cohort studies.

Fig 3 presents multivariable-adjusted risk estimates of pancreatic cancer from each cohort and all cohorts combined. Three of the 6 cohorts (accounting for 36.1% of total cases) reported an inverse association between vitamin C intake and risk of pancreatic cancer (RR ranged between 0.79 and 0.91), but none showed statistical significance. Overall, the summary RR for the highest-versus-lowest intake of vitamin C was 0.93 (95% CI: 0.78–1.11), with no evidence of heterogeneity (*P*_heterogeneity_ = 0.95, *I*^2^ = 0%). The null association persisted in the subgroup analysis (Table 2). Supplementary vitamin C showed a nonsignificant inverse association with pancreatic cancer (RR = 0.83, 95% CI: 0.64–1.07), but only 2 studies were included in the analysis. Sex-specific analysis was not performed due to limited studies. Dose-response meta-analysis of all cohorts showed a RR of 0.97 (95% CI: 0.89–1.05) for each 100-mg/day increment in vitamin C intake.

**Table 2:** Results of stratified analysis for cohort studies.

	*N*	RR (95% CI)	*P* for heterogeneity	*I*^2^ (%)	*P* for differences
Area					
North America	3	0.86 (0.66–1.13)	0.80	0	0.49
Europe	3	0.99 (0.78–1.25)	0.94	0	
Length of follow-up					
≥10 years	3	0.98 (0.78–1.24)	0.96	0	0.51
<10 years	3	0.86 (0.65–1.14)	0.76	0	
No. of cases					
≥200	2	1.00 (0.78–1.28)	0.97	0	0.49
<200	4	0.87 (0.68–1.12)	0.90	0	
Source of vitamin C					
Diet	5	0.92 (0.73–1.16)	0.97	0	0.56
Supplement	2	0.83 (0.64–1.07)	0.96	0	

### Meta-analysis of all studies

A combined analysis of the 20 observational studies (4,958 cancers) yielded a summary OR of 0.66 (95% CI: 0.58–0.75), with moderate heterogeneity among studies (*P*_heterogeneity_ = 0.05, *I*^2^ = 35.8%). Meta-regression analysis suggested a statistically significant difference in the results between case-control and cohort studies (*P*_difference_<0.001).

### Publication bias

Egger’s test indicated possible publication bias in the meta-analyses of case-control studies (*P*_Egger_ = 0.07) and all studies (*P*_Egger_ = 0.06), but notin the analysis of cohort studies (*P*_Egger_ = 0.61). Begg’s test excluded such a bias (*P*_Begg_>0.20).

## Discussion

In this meta-analysis of published observational studies that involved nearly 5,000 pancreatic cancers, high vitamin C intake was significantly associated with a 42% reduction in the risk of pancreatic cancer in case-control studies, whereas no association was found in cohort studies.

Fruit and vegetables are a rich source of dietary vitamin C, and have long been suspected to prevent several cancers including pancreatic cancer[8]. However, recent cumulative evidence showed that the protection of fruit and vegetables on pancreatic cancer was observed in case-control studies but not in cohort studies[39], which agreed with the findings of this meta-analysis.

The considerable differences between results from case-control studies and those from cohort studies may be attributed to several reasons. There was a suggestion of publication bias in case-control studies, which indicated that some small studies or studies with null effects may have been unpublished. However, such a bias was unlikely to fully explain why especially case-control studies showed an inverse association. It is more likely that the distinct findings were due to differences in methodologies they applied. Case-control studies relative to cohort studies are more prone to biases such as recall bias and selection bias. For instance, those foods rich in vitamin C (e.g., fruit and vegetables) have been widely considered beneficial for public health and also for cancer prevention. In this condition, patients with pancreatic cancer probably underreported their dietary intakes of these foods, leading to an overestimation of the association. Further, the temporal sequence between exposure and outcome is difficult to determine in case-control studies. In other words, cases may have altered their dietary intakes after the diagnosis of cancer. In addition, potential selection bias also merits a particular consideration when studying pancreatic cancer, a malignancy characterized by high fatality rates. On the other hand, both case-control and cohort studies are subject to exposure misclassification when investigating dietary factors and health outcomes, because they mostly used food frequency questionnaires to collect diet information. For cohort studies that generally made a single measurement of dietary intakes at baseline, additional misclassification of exposure could occur because participants may change their diet habits during a long-term follow-up period.

Circulating vitamin C concentrations represent a better indicator of vitamin C status than dietary intakes. However, current evidence linking circulating vitamin C to pancreatic cancer risk has been limited and inconclusive. Results from the European Prospective Investigation into Cancer and Nutrition (EPIC)-Norfolk study[35] showed a strong inverse association between serum vitamin C and pancreatic cancer during 17 years of follow-up (RR for inter-quartile comparison = 0.42, 95% CI: 0.20–0.91, 76 cancers). Conversely, a more recent case-control study nested in the whole EPIC study contradicted the previous findings (RR for inter-quartile comparison = 0.91, 95% CI: 0.55–1.51, 442 cancers)[12].

Long-term, double-blind, randomized controlled trials (RCTs) provide the most robust estimates of causal effects. However, such RCTs examining the effect vitamin C supplement on pancreatic cancer are also limited. A Cochrane systematic review[40] reported a null effect, but only 1 trial of vitamin C supplementation (combined with vitamin E and beta-carotene) and pancreatic cancer was included. The Physicians’ Health Study (PHS) II randomized trial[41] showed a nonsignificant 14% reduction in pancreatic cancer death (50 deaths) associated with vitamin C supplement after a mean follow-up duration of 8.0 years (RR = 0.86, 95% CI: 0.49–1.49). Subsequently, a posttrial observation[42] with additional 3.8 years of follow up strengthened the RR to be 0.50 (95% CI: 0.22, 1.18). This observation might raise the problem of a low statistical power resulting from short durations in these RCTs. Although well-designed RCTs provide the best evidence, the trials are expensive and only the most promising candidate can be tested in such a way. Given these null findings and possibly increased mortality associated with antioxidants supplements in clinical trials[40], it appears imprudent to specifically investigate the effects of vitamin C supplement on the prevention of pancreatic cancer in large RCTs before the possible benefits are consistently showed in large prospective observational studies.

This meta-analysis comprehensively quantified current evidence from published case-control and cohort studies comprising nearly 5,000 cases of pancreatic cancer and therefore largely strengthened the power of the analyses. However, we also acknowledged that there were several limitations to our study. Our results may have been affected by residual or unmeasured confounders because higher dietary vitamin C often positively corrected with better lifestyle patterns. What is more, as was discussed above, potential methodological flaws inherit in the original studies may bias our findings towards either direction. In addition, since the meta-analysis is on the basis of published literature, our results, in particular those from case-control studies may have been affected by publication bias.

In sum, this meta-analysis showed a significant inverse association between vitamin C intake and risk of pancreatic cancer in case-control studies but not in prospective cohort studies. Thus, there is insufficient evidence to conclude any relationship between vitamin C intake and risk of pancreatic cancer.

## Supporting Information

S1 PRISMA Checklist PRISMA Checklist. (DOCX)

S1 Table Characteristics of the included case-control studies of vitamin C intake and risk of pancreatic cancer. (DOCX)

S2 Table Characteristics of the included cohort studies of vitamin C intake and risk of pancreatic cancer. (DOCX)
