# Supplementary material for: Vitamin C Intake and Pancreatic Cancer Risk: A Meta-Analysis of Published Case-Control and Cohort Studies
Source: PLoS One. 2016 Feb 9;11(2):e0148816. doi: 10.1371/journal.pone.0148816 (PMC4747545; doi:10.1371/journal.pone.0148816)
Supplement: S1 Table — (DOCX) [file pone.0148816.s002.docx]

| Author, year | Country | Sources of control | Sex | No. of cases/controls | Sources of vitamin C | Vitamin C intake | OR（95% CI） | Variables |
| --- | --- | --- | --- | --- | --- | --- | --- | --- |
| Falk, 1988 | USA | Hospital | M/F | 363/1234 | Diet | ≥148.5 (T3) vs. 66.7 (T1) mg/d | 0.38 (0.24-0.61) (M)  0.55 (0.31-0.96) (F) | Age, race, smoking, residence, and history of diabetes. |
| Howe, 1990 | Canada | Population | M/F | 249/505 | Diet | Q4 vs. Q1  (inter-quartile range: 226 mg) | 0.81 (0.51-1.30) | Age, sex, smoking, dietary fiber, and energy intake. |
| Baghurst, 1991 | Australia | Population | M/F | 104/253 | Diet | Q4 vs. Q1 | 0.46 (0.23-0.94) | Age, sex, smoking, alcohol, and energy intake. |
| Bueno de Mesquita, 1991 | The Netherlands | Population | M/F | 164/480 | Diet | Q5 vs. Q1 | 0.75 (0.47-1.19) | Age, sex, response status, smoking, and  energy intake. |
| Ghadirian, 1991 | Canada | Population | M/F | 179/239 | Diet | Q4 vs. Q1 | 0.71 (0.34-1.23)  1.06 (0.58-1.94) (M)  0.47 (0.22-0.98) (F) | Age, sex, smoking, response status, and  energy intake. |
| Zatonski, 1991 | Poland | Population | M/F | 110/195 | Diet | Q4 vs. Q1 | 0.37 (0.13-0.99) | Age, sex, smoking, residence, and energy intake. |
| Olsen, 1991 | USA | Population | M | 212/220 | Diet | Q4 vs. Q1 | 0.50 (0.30-0.90) | Age, smoking, alcohol, education history of diabetes, and energy intake. |
| Ji, 1995 | China | Population | M/F | 451/1552 | Diet | ≥57.2 (Q4) vs. ≤22.9 (Q1) mg/d (M)  ≥42.4 (Q4) vs. ≤28.9 (Q1) mg/d (F) | 0.53 (0.33-0.84) (M)  0.66 (0.37-1.20) (F) | Age, smoking, income, green tea (F only), response status, and energy intake |
| Silverman, 1998 ^a^ | USA | Population | F | 213/747 | Supplement | Ever vs. never | 0.50 (0.30-0.90) | Age, BMI, smoking, alcohol, energy intake, race, area, diabetes, cholecystectomy, and marital status. |
| Lin, 2005 | Japan | Population | M/F | 109/218 | Diet | ≥57.2 (T3) vs. ≤22.9 (T1) mg/d | 0.45 (0.22-0.94) | Age, sex, smoking, and residence. |
| Anderson, 2009 | Canada | Population | M/F | 422/312 | Supplement | Regularly vs. not regularly^b^ | 0.71 (0.51-1.00) | Age. |
| Gong, 2010 | USA | Population | M/F | 532/2525 | Total  Diet  Supplement | ≥712 (Q4) vs.＜142 (Q1) mg/d  ≥191 (Q4) vs.＜102 (Q1) mg/d  ＞450 (Q4) vs.＜142 (Q1) mg/d | 0.69 (0.51-0.94)  0.94 (0.70-1.30)  0.68 (0.52-0.87) | Age, sex, BMI, race, education, diabetes, smoking, alcohol, physical activity, vitamin supplement (for dietary intake), and energy intake. |
| Bravi, 2011 | Italy | Hospital | M/F | 326/652 | Diet | Q5 vs. Q1 (mean: 149.9 mg/d) | 0.44 (0.27-0.73)  0.80 (0.63-1.02) (M)  0.63 (0.47-0.86) (F) | Age, sex, BMI, smoking, education, diabetes, study center, year of interview, and energy intake. |
| Jansen, 2013 | USA | Hospital | M/F | 384/983 | Diet  Supplement | 116.58 (M) or 140.55 (F) (Q5) vs.32.79 (M) or 40.95 (F) (Q1) mg/1000kcal  60 vs. 0 mg/d (the same for M/F) | 0.51 (0.34-0.76)  0.77 (0.59-1.00) | Age, sex, BMI, smoking, alcohol, and energy intake. |

S1 Table. Characteristics of the included case-control studies of vitamin C intake and risk of pancreatic cancer.

BMI, body mass index; d, day; F, female; M, male; Q, quartile/quintile; T, tertile.

^a^ the study included both sexes but only the female data for vitamin C supplement could be included in the meta-analysis.

^b^ Regular supplementation was defined as taking vitamin C supplements at least once per week for at least 1 year.
