# Supplementary material for: Vitamin C Intake and Pancreatic Cancer Risk: A Meta-Analysis of Published Case-Control and Cohort Studies
Source: PLoS One. 2016 Feb 9;11(2):e0148816. doi: 10.1371/journal.pone.0148816 (PMC4747545; doi:10.1371/journal.pone.0148816)
Supplement: S2 Table — (DOCX) [file pone.0148816.s003.docx]

S2 Table Characteristics of the included cohort studies of vitamin C intake and risk of pancreatic cancer

| Author, year | Country | Follow-up years | Sex | No. of cases/ participants | Sources of vitamin C | Vitamin C intake | RR（95% CI） | Variables |
| --- | --- | --- | --- | --- | --- | --- | --- | --- |
| Shibata, 1994 | USA | 9 | M/F | 65/13,979 | Diet | ≥220 (T3) vs. <150 (T1) mg/d | 0.79 (0.44-1.43) | Age, sex, and smoking. |
| Stolzenberg-Solomon, 2002 | Finland | 10.2 | M | 163/27,111 | Diet | >123 (Q5) vs.≤62 (Q1) mg/d | 0.91 (0.52-1.59) | Age, smoking, and intakes of folate and energy. |
| Inoue-Choi, 2011 | USA | 21 | F | 256/34,642 | Diet | 678.55 (Q5) vs. 82.4 (Q1) mg/d | 0.99 (0.56-1.49) | Age, race, education, smoking, alcohol, physical activity. |
| Heinen, 2011 | The Netherlands | 16.3 | M/F | 423/120,852 | Total  Supplement | 130.1 (M) or 140.5 (F) (T3) vs.  52.1 (M) or 58.9 (F) (T1) mg/d  Yes vs. no | 1.00 (0.74-1.33)  0.83 (0.59-1.18) | Age, sex, BMI, smoking, family history of pancreatic cancer, history of diabetes, and intakes of energy, red meat, coffee, and alcohol. |
| Han, 2013 | USA | 7.1 | M/F | 184/77,446 | Total  Diet  Supplement | 347.29-2629.64 (T3) vs. 0.57-137.20 (T1) mg/d  135.01-1854.97 (T3) vs. 0.57-76.64 (T1) mg/d  149.00-1750.00 vs. nonuser | 0.82 (0.56-1.21)  0.89 (0.58-1.35)  0.82 (0.56-1.19) | Age, sex, ethnicity, education, BMI, physical activity, smoking, family history of pancreatic cancer, history of diabetes, and intakes of energy and alcohol. |
| Banim, 2013 | UK | 8.6 | M/F | 49/3970 | Diet | 111.5-654.8 (Q4) vs. <51.3 (Q1) mg/d | 1.04 (0.58-1.85) | Age, sex, BMI, smoking, history of diabetes, and intakes of energy and supplementary vitamin C. |

BMI, body mass index; d, day; F, female; M, male; Q, quartile/quintile; T, tertile.
